# Supplementary material for: Transition from sexuality to androgenesis through a meiotic modification during spermatogenesis in freshwater Corbicula clams
Source: PLoS One. 2024 Nov 26;19(11):e0313753. doi: 10.1371/journal.pone.0313753 (PMC11594415; doi:10.1371/journal.pone.0313753)
Supplement: S1 Fig — A. Percentage of female and male follicles. Mixed regions of the tubules were omitted for more clarity. B. Spermatogenesis cycle. The tubule stages refer to S1 Table. For each spermatogenesis stage, the number of observations varies between 0 (not observed) and 5 (observed in all individuals). (DOCX) [file pone.0313753.s001.docx]

**A**

**B**

**Figure S1:** **progression of gametogenesis throughout the year in mature hermaphroditic *Corbicula* sp. form A/R individuals (n=60). A.** Percentage of female and male follicles. Mixed regions of the tubules were omitted for more clarity. **B.** Spermatogenesis cycle. For each spermatogenesis stage, the number of observations varies between 0 (not observed) and 5 (observed in all individuals).
