# Supplementary material for: Transition from sexuality to androgenesis through a meiotic modification during spermatogenesis in freshwater Corbicula clams
Source: PLoS One. 2024 Nov 26;19(11):e0313753. doi: 10.1371/journal.pone.0313753 (PMC11594415; doi:10.1371/journal.pone.0313753)
Supplement: S1 File — (DOCX) [file pone.0313753.s002.docx]

**Supplemental information 1: Feulgen staining protocol**

| **Reagents** | **Durations** |
| --- | --- |
| **Toluene** | 3 x 10’ |
| **Ethanol-formol (9/1, v/v)** | 15’ |
| **Ethanol 70%** | 5’ |
| **Distilled water** | rapid rinse |
| **HCL 5-6N** | 12’ |
| **Tap water** | 30’-1h |
| **Schiff** | 45’-2h |
| **Sodium bisulfite 0.5%** | 10’ |
| **Tap water** | 30’-1h |
| **Distilled water** | rapid rinse |
| **Ethanol 95** | 1’ |
| **Ethanol 100** | 1’ |
| **Toluene** | 2 x 5’ |
