# Supplementary material for: Transition from sexuality to androgenesis through a meiotic modification during spermatogenesis in freshwater Corbicula clams
Source: PLoS One. 2024 Nov 26;19(11):e0313753. doi: 10.1371/journal.pone.0313753 (PMC11594415; doi:10.1371/journal.pone.0313753)
Supplement: S1 Table — (DOCX) [file pone.0313753.s003.docx]

| **Stage 0** | Undifferentiated and quiescent stage of spermatogenesis.  The tubules appear as small and condensed mass only constituted of the germinal epithelium and undifferentiated germ cells. | No picture available |
| --- | --- | --- |
| **Stage I** | Early development stage.  Germ cells are dividing and differentiation starts. Four different cell types are visible: putative stem cells, spermatogonia, spermatocytes and spermatids (Fig. 2). No spermatozoa are present. | 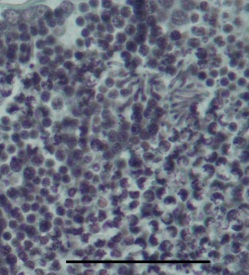 |
| **Stage II** | This stage only differs from stage I by the presence of developing spermatozoa. | 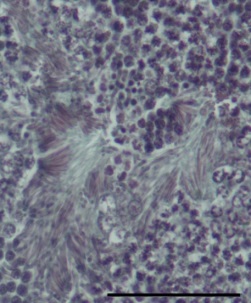 |
| **Stage III** | Late development stage of male follicles.  Spermatozoa are numerous and organized into rosettes. All other germ cell types are still present but their abundance is reduced. | 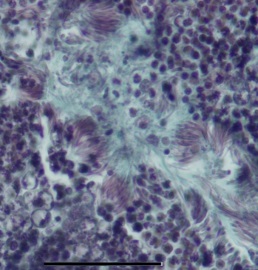 |
| **Stage IV** | Active discharge stage of fully mature gametes. Tubules appear as empty or with a lumina full of intermingling spermatozoa being expulsed. | 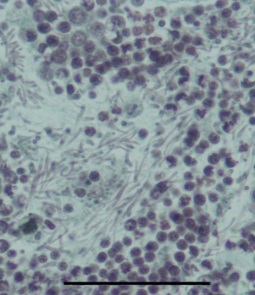 |
| **Stage V** | Late stage of spermatogenesis.  Some mature germ cells are not completely spawned and residual spermatozoa may be numerous. Tubules are loose and disorganized as remaining germ cells detach from the germinal lining. Lysis is important as revealed by the presence of macrophages. | 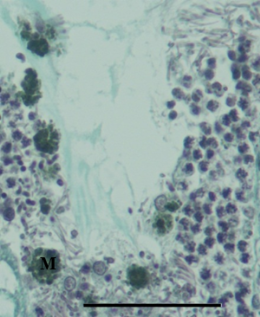 |

**Table S1: Spermatogenesis stages description of *Corbicula* sp. form A/R tubules.**

Scale bar = 60 µm, M = Macrophage. Prenant-Gabe Trichrome staining.
