# Supplementary material for: Transition from sexuality to androgenesis through a meiotic modification during spermatogenesis in freshwater Corbicula clams
Source: PLoS One. 2024 Nov 26;19(11):e0313753. doi: 10.1371/journal.pone.0313753 (PMC11594415; doi:10.1371/journal.pone.0313753)
Supplement: Attachment — Submitted filename: 20240930 Response to reviewers Final.docx [file pone.0313753.s004.docx]

Response to reviewers

**We are thankful to the editor and reviewers for their careful revision of our manuscript. In the present letter, the reviewer’s comments are displayed in italic, while the authors’ responses are in bold. We also wish to draw your attention on a small title modification: “Transition from sexuality to androgenesis through a meiotic modification in freshwater *Corbicula* clams” is now “Transition from sexuality to androgenesis through a meiotic modification during spermatogenesis in freshwater *Corbicula* clams”. Note that the line numbers in this document refer to the revised manuscript with track changes and might be slightly different in the unmarked version.**

*Reviewer #1: In their paper, Etoundi and colleagues present the first study evaluating the potential mechanism of spermatogenesis in obligate androgenetic species. The main objectives of the paper are to 1) understand how to produce unreduced gametes through spermatogenesis and 2) understand why androgenesis is repeatedly found in Corbicula clams. To achieve this, they successfully employed a computer-assisted image cytometry technique in a non-model organism, the Corbicula clam. They used image cytometry to distinguish among three mechanisms (abortive step of meiosis, fusion, or chromosome duplication). They demonstrated that:

- C. sandai and C. japonica :
o Both sexual species, C. japonica and C. sandai, are diploid and mainly produce reduced spermatozoa.
o Natural sexual species produce unreduced spermatozoa, with C. sandai representing 44.76 and 33.33% of unreduced spermatozoa and spermatids. This indicates a permissive capacity for reproducing asexually. The modification of meiosis leading to the formation of these unreduced is not identified, but the data excluded pre-meiotic endoduplication.
o In both C. sandai and C. japonica, the existence of unreduced spermatozoa correlates with the observation of biflagellate spermatozoa.

- Corbicula form A/R:
o The data shows different amounts of genetic material in somatic tissue (1C, 2C, 3C, 4C and 6C), indicating distinct levels of ploidy. These different ploidy states could belong to the same individual or represent variations in ploidy among individuals of the same species.
o The absence of 12C content clearly excludes pre-meiotic duplication during spermatogenesis.
o Furthermore, due to the high level of heterozygosity, abortive meiosis I is a more parsimonious mechanism than abortive meiosis II.


Minor comments:

- Were you able to distinguish the 2C biflagellate from the two overlapping 1C spermatozoa, or are they considered debris?*

**Unfortunately, the Feulgen approach did not allow distinguishing the flagella, that are particularly thin. This staining is well-adapted for DNA staining but makes other structures hardly visible.** *- What is the sex-determining system of Corbicula form A/R? Could it confirm your results, i.e., abortive meiosis I?*

**Thank you for asking this question. Bivalve sex-determining mechanisms were quite recently reviewed (here:** [**https://doi.org/10.1007/978-3-319-94139-4_6**](https://doi.org/10.1007/978-3-319-94139-4_6)**), with a short discussion on *Corbicula* androgenesis. Unfortunately, the sex-determining system in *Corbicula* is still unknown, and because our discussion section is already long (as noticed by reviewer #2), we have chosen not to develop a discussion on sex determination in our manuscript. However, this is still a good point to be mentioned. We have added a short sentence on this topic in lines 375-376.** *- Do you observe embryonic lethality?*

**This is a good question. To our knowledge, this was never studied in *Corbicula*. This might be considered for future studies.** *- in Corbicula form A/R:
o In the case of triploid chromosomes where pre-meiotic endoreplication does not occur, homologous chromosomes are unable to pair. Consequently, incomplete meiosis I is inevitable, or if meiosis is attempted, it will lead to incorrect segregation of the chromosomes.*

**This is a good remark. Indeed, meiosis I with an odd chromosome number would likely result in aneuploidy, which was never observed in *Corbicula* clams. This was discussed lines 356-358.** *o Is it possible that the observed 2C and 1C spermatozoa resulted from inequivalent segregation of homologous chromosomes during meiosis I, producing two products with either 2C or 1C content?*

**We formulated this hypothesis at some point, but it is likely not verified, so we have chosen not to include it in the manuscript. Mixed production of both 1C and 2C spermatozoa in high proportions was only observed in *C. sandai*, who is diploid. Moreover, inequivalent segregation of homologous chromosomes during meiosis I would most likely produce aneuploid products, except if a cytological mechanism allows one sister cell to receive only one chromosome from each set and the other sister cell to receive always two chromosomes from each set. Such mechanism was never demonstrated in *Corbicula*.** *o The somatic presents different ploidy states that could result from multiple fertilisations, leading to the development of mosaic animals.*

**Mosaicism is indeed not excluded in *Corbicula* clams, as discussed lines 385-389. However, these different ploidy states might also result from different individuals, as ploidy is variable in these clams, even within the same species.**

*Reviewer #2: The manuscript by Etoundi & al. analyses the origin of unreduced sperm production in an androgenetic Corbicula species. Androgenesis is a fascinating reproductive system in which the oocyte DNA is completely expelled at fertilisation. As a result, the zygote develops only from paternal DNA, maintaining diploidy if the sperm is unreduced.
Previous observations have shown the presence of biflagellate spermatozoa in an androgenetic Corbicula. Since the flagella is derived from a centrosome, which is inherited along with a nucleus in each sperm cell at the end of meiosis, the presence of two flagella per sperm cell suggests that the nuclei should also be present in two copies in the sperm cell.
However, this has not been verified, and the present study aims to confirm the DNA content of mature spermatozoa in androgenetic Corbicula species compared to normally sexual species.

The study is interesting and new. I have however a couple of important point to raise and several minor comments.

Major comments:
- It is necessary to give a clear explanation of the calibration of ploidy in this study. It is clear for the sexual species where the reduced spermatozoa can be directly assigned to 1C. What defines 1C in the androgenetic species? The rare cells with the lowest DNA content? The whole study and analysis is based on this, it is necessary to clarify this point.*

**We agree that distinct ploidy populations could not be retrieved as clearly for form A/R than for *C. sandai* and *C. japonica* (lines 299-300) and that the delimitations of the different cell populations based on the cell distribution is a bit arbitrary (line 301). However, a comparison between somatic and sperm cells in form A/R clearly show similar profiles, indicating that the sperm cells are unreduced. Moreover, this lineage is known for being mostly triploid, and our analysis of the cell distribution is consistent with this prior knowledge.** *- The androgenetic species studied does not originate from Lake Biwa, but from a river in Belgium. This is not explicitly stated in the main text, but only in the materials and methods. Although it doesn't change the conclusion of the study, it does change the discussion a bit, because since we don't know the origin of this Belgian species, it is more difficult to imagine that C. sandai serves as a reservoir for new androgenetic species. Please explain why the androgenetic species from Lake Biwa mentioned in the introduction has not been studied here.*

**The original aim of this study was to understand the androgenetic mechanism leading to unreduced sperm production of the most widespread invasive lineages in Belgium, namely *C.* sp. form A/R. To achieve this, sexual species had to be acquired from another continent, because no sexual species was ever recorded in the invaded area, *i.e.*, Europe and America. Sexual species from Asia were selected notably because the invasion likely originated from this continent. In a recent study (Vastrade *et al*. 2022,** [**https://peercommunityjournal.org/articles/10.24072/pcjournal.180/**](https://peercommunityjournal.org/articles/10.24072/pcjournal.180/)**), we have shown the occurrence of a substantial allele sharing between European lineages and Lake Biwa species. It is thus not that difficult to imagine that *C. sandai* serves as a reservoir for new androgenetic species, some of them being introduced in other continents.**
*- Please explicitly explain the link between biflagellate and unreduced sperm.*

**Thank you for highlighting that this point was not clear. It was clarified lines 123-125.** *- Conclusion of unreduced gamete being also found in C. sandai: could it come from a mistake in the assignment of cell types? For instance if spermatocytes and some spermatids are mixed up ? this should be included in the discussion as a possibility*

**Such confusion would be unlikely because of the characteristic morphology of mature spermatozoa compared to spermatogonia and spermatocytes, and because any doubtful cell was considered as debris. This is discussed lines 336-338.**
*- The discussion takes too long*

**The section that you point as unclear was significantly reduced, lines 477-492. If you detect other sections which would deserve to be reduced, please let us know.** *Minor comments:
- include Corbicula in the keywords*

**Good point, this was added line 39.** *-abstract: “by the production unreduced sperm”, OF is missing*

**Thank you, this was corrected line 27.** *-abstract: Rather than "a potential switch between sexuality and androgenesis", would you say that “the production of unreduced sperm in C. sandai may condition or provide the potential for the emergence of androgenesis”?*

**This is well written, but this suggestion would not reflect the transition from sexuality to androgenesis. We thus adapted it into: “The production of unreduced sperm may condition or provide the potential for the emergence of androgenesis in this sexual species.” See lines 32-34.** *-line 75: out of curiosity, in stick insects, if the system produces only males, does it mean there is fusion between X-bearing and Y-bearing sperm only ?*

**We thank you for your curiosity on androgenesis. The *Bacillus* stick insect sex-determining system in a X0 system, with XX females and X0 males. On one hand, the all-male androgenesis is likely achieved through fertilization by balanced unreduced spermatozoa. The offspring thus receive all chromosomes from the father, including the sexual chromosomes X0, resulting in an all-male progeny. On the other hand, fusion of two sperms result in a bisexual progeny. Note that this progeny is often biased to female (*e.g.*, 78% females in Tinti & Scali 1995,** [**https://doi.org/10.1002/jez.1402730208**](https://doi.org/10.1002/jez.1402730208)**), while theory (*e.g.*, Punnett square on sexual chromosomes from two X0 spermatozoa) would predict 67% males (with 00 individuals considered not viable). Genetic alone thus fails explaining the observed prevalence of females in this case, which might be due to selection.** *-line 97, please specify if these are self-fertilizing hermaphrodites or cross-fertilizing*

**This was specified lines 98 and 101.** *-line 109: not entirely clear because the defect in female meiosis does not lead to fusion of meiotic products: “as observed during oogenesis” is a bit misleading*

**The sentence (currently lines 111-113) was subdivided to clarify that the comparison to oogenesis is only about an aborted meiotic division.** *-line 159, a reference for the culture conditions is missing*

**A reference was added lines 164 and 667-669.**
*-line 177: Can you explain why you use the Feulgen stain and not the classic Dapi stain?*

**The Feulgen staining offers a stochiometric relationship between DNA content and staining intensity, which can be measured by image analysis with a high precision. This method was already optimized in Fafin-Lefevre *et al*. 2011 (**[**https://pubmed.ncbi.nlm.nih.gov/21980622/**](https://pubmed.ncbi.nlm.nih.gov/21980622/)**). The DAPI staining is less adapted for this kind of analysis.** *-line 236: more abundant than ? than male tissue ?*

**Indeed, this was specified line 241.** *-Figure 1: specify this is the theoretical expectation, not a result of this study (this could go in a supplementary figure, not as a main figure)*

**This was specified line 788. We have made the choice to leave it as a main figure because it is important for understanding the manuscript.**  *-Figure 2: The panel showing the SR region is of poor quality, this is a very important part of the study, this needs to be improved.*

**The figure was redone.** *-line 448: “these mechanisms” refer to what. ?pre and postmeiotic defects as described in plants ? the transition is unclear*

**This was specified line 457.** *-line 468: “suggest an evolutionary utility for such unsual gametes” ..but it could also simply reflect that meiosis is not perfect and often fails ..(see the many meiotic defects in female meiosis in mouse and humans for instance)*

**This claim was toned down lines 477-478.** *-line 468-471: improve, not entirely clear*

**This section was reworked and reduced lines 477-492.** *-line 479: the sentence is a bit unclear: “unreduced gametes in asexual confer an adaptative potential”: unreduced gametes confer an adaptative potential for the emergence of asexuality (because this is a prerequisite) ..but for an established asexual lineage, I don’t see the adaptative advantage.*

**Thank you for this remark, highlighting that this section was indeed unclear. The adaptive potential can be conferred through polyploidy. We hope this section is clearer now.**
*-line 481: there have been other cytological studies prior to this study, in particular those that have described the biflagellate sperm in Corbicula.*

**This claim was toned down line 494.**
