# Supplementary material for: Transition from sexuality to androgenesis through a meiotic modification during spermatogenesis in freshwater Corbicula clams
Source: PLoS One. 2024 Nov 26;19(11):e0313753. doi: 10.1371/journal.pone.0313753 (PMC11594415; doi:10.1371/journal.pone.0313753)
Supplement: Attachment — Submitted filename: 20241024 Response to reviewers.docx [file pone.0313753.s005.docx]

Response to reviewers

**We are thankful to the editor and reviewers for their careful revision of our manuscript. In the present letter, the reviewer’s comments are displayed in italic, while the authors’ responses are in bold. Note that the line numbers in this document refer to the revised manuscript with track changes and might be slightly different in the unmarked version.**

PLOS ONE

Dear Dr. VASTRADE,

Thank you for submitting your manuscript to PLOS ONE. After careful consideration, we feel that it has merit but does not fully meet PLOS ONE’s publication criteria as it currently stands. Therefore, we invite you to submit a revised version of the manuscript that addresses the points raised during the review process.

You should revise your manuscript based on reviewers' suggestions, questions and comments, not just answer their questions in the letter.

**The few points raised by the reviewer #2 have been addressed in the present version of the manuscript, based on the answers provided in the previous letter.**

Reviewer #2: I am satisfied with all the answers given in the letter. I regret, however, that some answers and clarifications have not been incorporated into the manuscript, in particular :
-explanation on the calibration of ploidy for the A/R form

**This clarification was incorporated in the manuscript lines 308-309 and 311.**
-the rationale for studying the sexual form in lake Biwa, while the androgenetic species is from Belgium

**This clarification was incorporated in the manuscript lines 159-164.**
-the rationale for using the Feulgen staining and not DAPI: simply state somewhere in the text that DAPI is less adapted in this case …

**This was mentioned line 189.**
